# Sunrouge, an Anthocyanin‐Rich Green Tea Cultivar, Attenuates Fat Accumulation and Obesity‐Related Metabolic Abnormalities in Western Diet‐Fed Mice

**DOI:** 10.1002/fsn3.72186

**Published:** 2026-08-26

**Authors:** Chika Ando, Kyohei Furukawa, Shunichi Someya, Yongshou Yang, Maki Igarashi, Seigo Yamada, Huijuan Jia, Hisanori Kato

**Affiliations:** ^1^ Graduate School of Agricultural and Life Sciences The University of Tokyo Tokyo Japan; ^2^ Animal Nutrition, Graduate School of Bioagricultural Sciences Nagoya University Nagoya Japan; ^3^ School of Life Sciences Anhui University Hefei China; ^4^ Division of Epidemiology National Cancer Center Institute for Cancer Control Tokyo Japan; ^5^ AZUMA CHEMICAL Co. Ltd. Chiyoda‐ku Tokyo Japan; ^6^ Nutritional Biochemistry, Department of Applied Nutrition, Undergraduate School of Nutrition Sciences Japan Nutrition University Sakado Japan

**Keywords:** anthocyanin, catechin, obesity, Sunrouge

## Abstract

Obesity is a major risk factor for the development of lifestyle‐related diseases worldwide. Green tea (GT) and catechins have been reported to ameliorate obesity‐related phenotypes, whereas the metabolic effects of Sunrouge (SR), a GT cultivar containing both catechins and anthocyanins, remain insufficiently characterized. In this study, we investigated the effects of long‐term SR intake in Western diet (WD)‐fed male C57BL/6J mice and examined the effects of epigallocatechin gallate (EGCG) and delphinidin‐3‐*O*‐galactoside (D3Ga), representative SR polyphenols, in HepG2 cells. In WD‐fed mice, both SR and GT attenuated adipose tissue mass, hepatic triglyceride (TG) and nonesterified fatty acid (NEFA) accumulation, and improved glucose tolerance in the intraperitoneal glucose tolerance test. SR additionally reduced plasma and hepatic total cholesterol and increased fasting plasma active glucagon‐like peptide‐1 concentrations. SR also altered cecal organic acid profiles and intestinal microbiota composition. In HepG2 cells, D3Ga significantly reduced NEFA‐induced intracellular TG accumulation, and co‐treatment with EGCG and D3Ga increased *CPT1* expression. These findings suggest that SR, like GT, attenuates diet‐induced fat accumulation and several obesity‐related metabolic abnormalities. SR also affected cholesterol metabolism, fasting plasma active GLP‐1 levels, and gut microbial profiles, suggesting a partly distinct metabolic profile from GT. This study provides new insights into the potential metabolic benefits of anthocyanin‐containing GT cultivars.

## Graphical abstract

Proposed metabolic effects of Sunrouge tea based on the findings of the present study. Sunrouge contains both catechins, represented by epigallocatechin gallate (EGCG), and anthocyanins, represented by delphinidin‐3‐*O*‐galactoside (D3Ga). Long‐term intake of Sunrouge attenuated diet‐induced fat accumulation and obesity‐related metabolic abnormalities, accompanied by inhibition of pancreatic lipase activity, altered expression of lipid metabolism‐related genes, changes in the intestinal microbiota, and increased fasting plasma active glucagon‐like peptide‐1 (GLP‐1) levels. These changes were associated with improved lipid metabolism, reduced plasma and hepatic total cholesterol levels, and attenuated glucose excursion during the intraperitoneal glucose tolerance test (IPGTT).

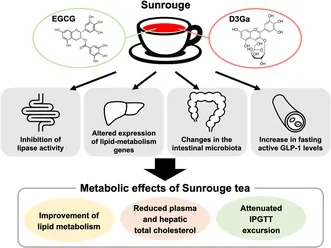

**Abbreviations**
AMPK: adenosine monophosphate‐activated protein kinase
AUC: area under the curve
CON: control
D3Ga: delphinidin‐3‐*O*‐galactoside
EGCG: epigallocatechin gallate
GLP‐1: glucagon‐like peptide‐1
GT: green tea
IPGTT: intraperitoneal glucose tolerance test
ITT: insulin tolerance test
NEFA: nonesterified fatty acid
OGTT: oral glucose tolerance test
SR: Sunrouge
TC: total cholesterol
TG: triglyceride
WD: Western diet

## 1 Introduction

The prevalence of obesity among both children and adults is rapidly increasing worldwide. Obesity results from an imbalance between calorie intake and expenditure and is characterized by various physiological abnormalities, including increased body weight, adiposity, dyslipidemia, hyperglycemia, and insulin resistance. These alterations accelerate the onset of several noncommunicable diseases, including type 2 diabetes, cardiovascular disease, and cancer (Antwi 2023; Calle and Kaaks 2004; Piché et al. 2018). Therefore, the development of nutritional strategies for obesity prevention and treatment is essential for maintaining quality of life (Yoo 2018).

Excessive dietary fat intake results in increased intestinal absorption of fatty acids (FAs), thereby promoting the hepatic synthesis and accumulation of triglycerides (TGs) (Lundsgaard et al. 2023). Excess TG deposition in the liver is strongly associated with oxidative stress, inflammation, and cellular dysfunction (Ezhilarasan 2018; Klaunig et al. 2011; Cheng et al. 2008). In addition, the gut microbiota is increasingly recognized as a key factor influencing metabolic diseases, and several microbial genera have been linked to disease states (Apalowo et al. 2024). Microbial metabolites, including short‐chain fatty acids (SCFAs), play important roles in host metabolism by influencing gastrointestinal hormone secretion, appetite regulation, and insulin homeostasis.

Green tea (GT), produced from the leaves of *Camellia sinensis* , has been studied extensively because it contains abundant catechins such as epicatechin, epigallocatechin, epicatechin gallate, and epigallocatechin gallate (EGCG) (Meyer et al. 2023). GT and catechins have been reported to improve obesity‐related and other metabolic abnormalities, including dyslipidemia and impaired glucose metabolism (Li et al. 2018; Xu et al. 2020; Yang et al. 2016; Tang et al. 2021). In particular, EGCG has been shown to suppress fat accumulation and improve glucose tolerance (Moon et al. 2007; Park et al. 2020; Talebi et al. 2021).

Sunrouge (SR) is a GT cultivar generated by crossbreeding *Camellia sinensis* with *Camellia taliensis* (Saito et al. 2011). SR is distinct from traditional GT because it contains both catechins and anthocyanins—such as delphinidin‐3‐*O*‐galactoside (D3Ga), delphinidin‐3‐*O*‐glucoside, cyanidin‐3‐*O*‐galactoside, and cyanidin‐3‐*O*‐glucoside; whereas typical GT contains catechins but no detectable anthocyanins. Anthocyanins are also known to exert bioactive effects, including the amelioration of metabolic disorders (Yang et al. 2017). Therefore, SR may exert metabolic effects distinct from those of conventional GT.

Previous animal studies have suggested that SR may ameliorate colitis (Akiyama et al. 2012), inhibit pancreatic lipase activity (Shirai 2017), and improve postprandial glucose regulation (Wasai et al. 2018). However, the long‐term effects of SR on Western diet (WD)‐induced metabolic abnormalities have not been fully investigated. Therefore, in the present study, we examined: (1) the effects of long‐term SR intake on obesity‐related metabolic phenotypes in WD‐fed mice, and (2) the effects of EGCG and D3Ga, two representative bioactive components of SR, on lipid accumulation in HepG2 cells.

## 2 Materials and Methods

### 2.1 Materials

SR (HINOAKANE) and GT powders used in this study were commercially available processed tea products provided by AZUMA CHEMICAL Co. Ltd. (Tokyo, Japan) and ITO EN Ltd. (Tokyo, Japan), respectively. The SR powder was provided as HINOAKANE, a commercial SR tea product, whereas the GT powder was provided as Oi Ocha Instant Bold Green Tea with Matcha, a commercial instant GT product. Catechin and anthocyanin contents were analyzed by Japan Food Research Laboratories (Tokyo, Japan) using the same powder batches as those used in the animal study, confirming that SR contained both catechins and anthocyanins, whereas GT contained catechins only (Table S1).

### 2.2 Animal Experiments

Six‐week‐old male C57BL/6J mice (CLEA Japan Inc., Tokyo, Japan) were maintained under a 12‐h light/dark cycle (lights on 8:00–20:00), at 22°C ± 1°C and 60% ± 5% humidity. After 1 week acclimatization on a standard control diet, mice were allocated to four groups so that the mean initial body weight was comparable among the groups: control (CON; control diet, *n* = 9), WD (44% kcal from fat, *n* = 10), SR (WD supplemented with 3.5% SR powder, *n* = 11), and GT (WD supplemented with 3.5% GT powder, *n* = 10). Mice were housed individually and had ad libitum access to diet and water for 12 weeks. The ingredient composition of each diet is shown in Table S2. The supplementation level (3.5%) was determined using the human equivalent dose (HED) method (Reagan‐Shaw et al. 2008) to enable long‐term supplementation without compromising palatability. Food intake and total energy intake were monitored throughout the study (Figure S1). After 12 weeks, mice were fasted for 6 h and euthanized under isoflurane anesthesia. Blood, liver, hind limb muscle, mesenteric fat, epididymal fat, retroperitoneal fat, and cecal contents were collected and stored at −80°C. Blood was collected into heparinized tubes and immediately centrifuged (1000 g, 4°C, 15 min), and plasma was stored at −80°C.

### 2.3 Glucose and Insulin Tolerance Tests

Oral glucose tolerance tests (OGTT), intraperitoneal glucose tolerance tests (IPGTT), and insulin tolerance tests (ITT) were performed at Weeks 9, 10, and 11, respectively. For the OGTT, D‐glucose (2 g/kg body weight) in 0.9% saline (Kanto Chemical, Tokyo, Japan) was administered orally after a 16‐h fast. For the IPGTT, D‐glucose (1.5 g/kg) in 0.9% saline was injected intraperitoneally after a 6‐h fast. For the ITT, bovine insulin (0.75 U/kg; Sigma‐Aldrich) in 0.9% saline was injected intraperitoneally after a 4‐h fast. Blood glucose levels were measured at baseline (0 min) and 30, 60, 90, and 120 min after administration of glucose (OGTT/IPGTT) or insulin (ITT) solution using a Medisafe Fit blood glucose meter (Terumo Corporation, Tokyo, Japan). Areas under the curve (AUC) were calculated using the trapezoidal rule.

### 2.4 Biochemical Analyses

Plasma TG, total cholesterol (TC), glucose, nonesterified fatty acid (NEFA), active glucagon‐like peptide‐1 (GLP‐1), and insulin levels were measured using Triglyceride E‐Test Wako, Cholesterol E‐Test Wako, Glucose CII‐Test Wako, NEFA C‐Test Wako, GLP‐1 ELISA Kit Wako High Sensitivity Product (all from FUJIFILM Wako) and Mouse/Rat Insulin Assay Kit (Morinaga Institute of Biological Science Inc., Kanagawa, Japan), respectively, following manufacturers' instructions. Hepatic lipids were extracted by the Folch method (Folch et al. 1957), and TG, TC, and NEFA levels were measured using the same kits as those used for plasma.

### 2.5 Real‐Time Reverse Transcription Polymerase Chain Reaction (RT‐qPCR)

Total RNA was extracted from hepatic tissue and HepG2 cells using the NucleoSpin TriPrep kit (Takara Bio, Tokyo, Japan), and RNA concentration was measured using a NanoDrop ND‐1000 (Thermo Fisher Scientific). Complementary DNA was synthesized from total RNA using PrimeScript RT Master Mix (Perfect Real Time) (Takara Bio). Quantitative PCR was performed using SYBR Premix Ex Taq (Takara Bio) on a Thermal Cycler Dice Real Time System TP800 (Takara Bio) with the primers listed in Tables S3 and S4. mRNA levels were normalized to ribosomal protein S18 (*Rps18*) and presented as fold changes relative to the control group.

### 2.6 HPLC Analysis of Organic Acids in Cecal Contents

Organic acids in cecal contents were quantified as described by Tsukahara et al. (2014), with minor modifications. Briefly, 50 mg cecal content was mixed with 150 μL of distilled water and 22.5 μL of 12% perchloric acid on ice for 10 min. After centrifugation (13,000 *g*, 4°C, 10 min), the supernatant was filtered through a 0.45‐μm filter (Merck Millipore, Burlington, MA, USA). Samples (50 μL) were injected using an autosampler (SIL‐30 AC, Shimadzu). Two serial organic acid analysis columns (Shim‐pack SCR‐102H, 8.0 mm I.D. × 300 mm, 7.0 μm, Shimadzu) and a guard column (SCR‐102HG) were maintained at 50°C. Organic acids were separated isocratically at 0.8 mL/min with 5 mmol/L p‐toluenesulfonic acid and detected using an electrical conductivity detector (CDD‐10Avp, Shimadzu) after post‐column reaction with 5 mmol/L p‐toluenesulfonic acid, 20 mmol/L Bis‐Tris, and 100 μmol/L EDTA. Concentrations of organic acids are expressed as nmol/mg wet weight.

### 2.7 Microbiome Analysis

Microbiome analysis was performed using 16S rRNA gene amplicon sequencing of cecal contents (Yang et al. 2022). DNA was extracted from 50 mg of cecal content using the QIAamp Fast DNA Stool Mini kit (Qiagen, Hilden, Germany) according to the manufacturer's instructions. The V3–V4 region of the 16S rRNA gene was amplified with the primers 5′‐CCTACGGGNGGCWGCAG‐3′ and 5′‐GACTACHVGGGGTATCTAATCC‐3′. Amplicon size (~550 bp) was confirmed using an Agilent 2100 Bioanalyzer (Agilent Technologies, Santa Clara, CA, USA). PCR products were purified using Agencourt AMPure XP (Illumina, San Diego, CA, USA), re‐amplified for indexing, and purified again. DNA concentration was measured with the Qubit 4 fluorometer (Thermo Fisher Scientific). Equal volumes of each sample were pooled and sequenced on a MiSeq platform (Illumina). Raw sequencing data were processed using the DADA2 pipeline implemented in QIIME 2 (v2021.11) to generate amplicon sequence variant (ASV) tables. Taxonomic classification was performed against the SILVA database (v138). Alpha‐ and beta‐diversity analyses were performed after rarefaction to 39,412 reads per sample. This sampling depth corresponded to the minimum number of non‐chimeric reads retained among the analyzed samples. Alpha diversity (Faith's phylogenetic diversity, Shannon index, observed features, and Pielou's evenness) and beta diversity (weighted UniFrac, unweighted UniFrac, and Bray‐Curtis) were calculated with QIIME2 and visualized in R software (v4.3.2).

### 2.8 Assessment of Pancreatic Lipase Inhibition by SR and GT

Samples were prepared according to the method of Shirai (Shirai 2017) with minor modifications. Two grams of SR or GT powder was suspended in 50 mL of 60% aqueous acetone and stirred at room temperature for 15 h. After centrifugation (3500 *g*, 24°C, 10 min), the supernatant was evaporated under reduced pressure. The resulting residue was dissolved in 50% aqueous methanol. Pancreatic lipase activity was measured using Lipase Kit S (DS Pharma Biomedical, Osaka, Japan) according to the manufacturer's protocol, and absorbance was measured at 412 nm using a spectrophotometer (Thermo Fisher Scientific, Waltham, MA, USA). Orlistat (1 mg/mL) was used as a positive control for lipase inhibition.

### 2.9 Cell Culture and Intracellular Lipid Quantification

HepG2 cells were obtained from the National Center for Child Health and Development, Japan. Cells were cultured in Dulbecco's modified Eagle's medium (Sigma‐Aldrich, St. Louis, MO, USA) supplemented with 10% fetal bovine serum, 100 U/mL penicillin, and 100 μg/mL streptomycin at 37°C under 95% air/5% CO_2_. EGCG (Nagara Sciences, Gifu, Japan; ≥ 98% purity) and D3Ga (Sigma‐Aldrich; ≥ 97% purity) were prepared as 40 mM stock solutions in 100 mM HEPES (pH 7.2) and stored at −20°C. NEFAs were prepared as a 1:1 mixture of oleic acid and palmitic acid (Sigma‐Aldrich) as described previously (Lin et al. 2007). Cells were seeded in 60‐mm dishes at 6.0 × 10^5^ cells/dish in medium containing 10% FBS. At 60%–70% confluence, EGCG or D3Ga (20 or 50 μM) was added for 20 h. The medium was then replaced with FBS‐free medium with or without NEFAs, and cells were cultured for an additional 24 h. Intracellular lipids were extracted with 2‐propanol (Kanto Chemical CO. Ltd., Tokyo, Japan), and TG levels were measured using Triglyceride E‐Test Wako (FUJIFILM Wako) according to the manufacturer's instructions. DNA was extracted using NucleoSpin DNA Rapid Lyse (Takara Bio) and used to normalize intracellular lipid content.

### 2.10 Statistical Analysis

Data are expressed as mean ± standard error (SE). Longitudinal body weight data and the time‐course data from the OGTT, IPGTT, and ITT were analyzed using repeated‐measures two‐way analysis of variance (ANOVA), with group and time as factors. The group × time interaction was also evaluated, and the Geisser–Greenhouse correction was applied to the repeated‐measures analysis. Multiple comparisons were performed using Tukey's multiple‐comparisons test. For single‐time‐point measurements and area‐under‐the‐curve (AUC) values, one‐way ANOVA followed by the Tukey–Kramer post hoc test was used for multiple‐group comparisons. Beta‐diversity of the gut microbiota was assessed using permutational multivariate analysis of variance (PERMANOVA) based on Bray–Curtis distances. Taxon‐level comparisons were performed using the Kruskal–Wallis test followed by Dunn's multiple comparison test, with *p*‐values adjusted using the Benjamini–Hochberg method. Linear discriminant analysis effect size (LEfSe) analysis was additionally performed to identify bacterial taxa associated with each dietary group. Statistical significance was set at *p* < 0.05. For analyses involving false discovery rate correction, adjusted *p*‐values < 0.05 were considered statistically significant. Sample sizes for most analyses were *n* = 9 (CON), *n* = 10 (WD), *n* = 11 (SR), and *n* = 10 (GT). For organic acid and microbiome analyses, sample sizes were *n* = 9 (CON), *n* = 10 (WD), *n* = 9 (SR), and *n* = 10 (GT) because of limited sample volume.

## 3 Results

### 3.1 Effects of SR and GT Supplementation on WD‐Induced Obesity

**FIGURE 1:**
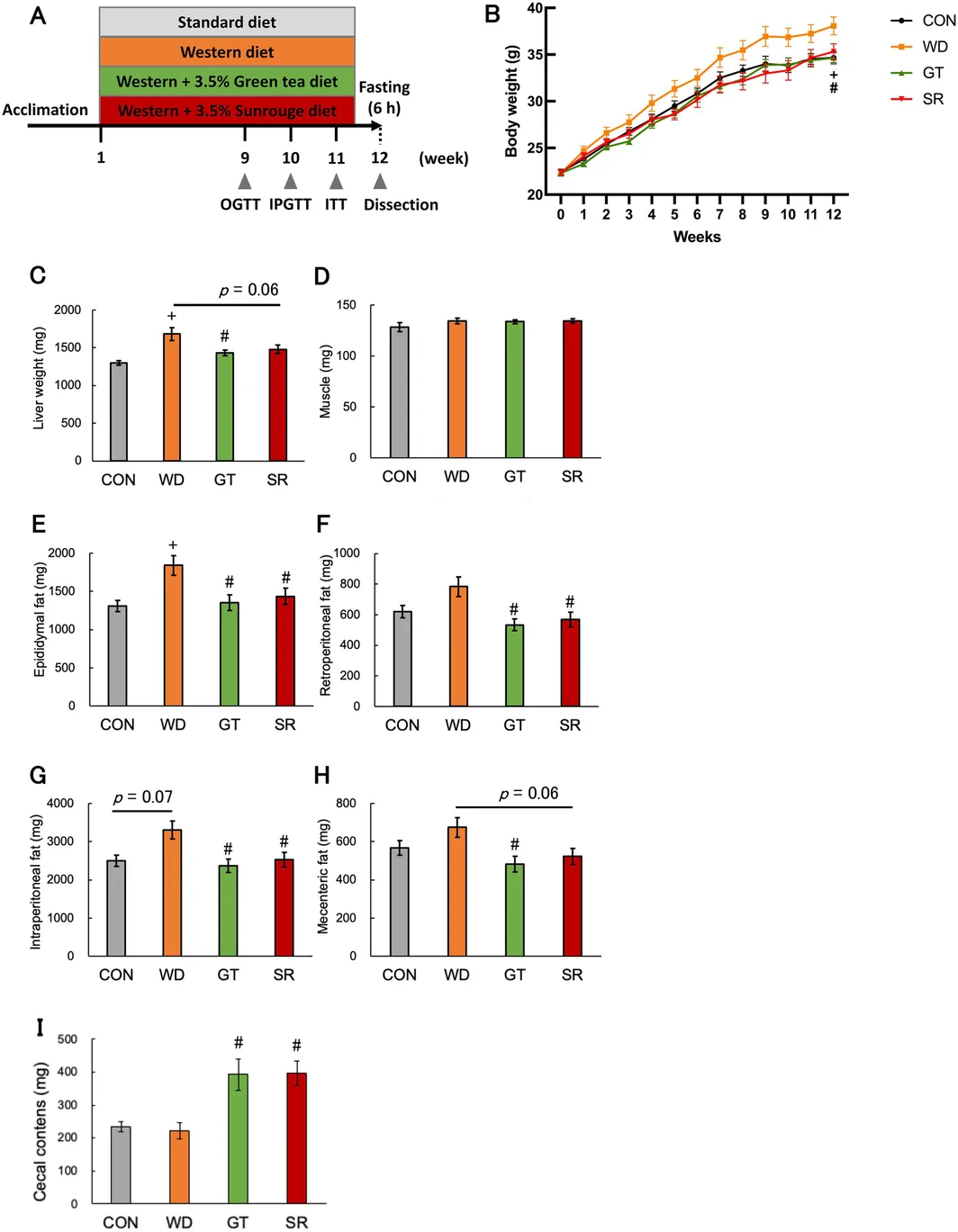
Effect of green tea (GT) and Sunrouge (SR) supplementation on the body composition in obese mice. (A) Schedule of the animal experiment. (B) Body weight during the feeding period. Symbols: + and ＃ indicate significant differences (*p* < 0.05) in control (CON) versus Western diet (WD), GT versus WD, and SR versus WD, respectively. (C–I) Liver weight, muscle weight, adipose depot weights (epididymal, retroperitoneal, intraperitoneal, mesenteric fat), and cecal content weight. Data are presented as mean ± SE (*n* = 9–11), ^+^ *p* < 0.05 versus CON, ^#^ *p* < 0.05 versus WD. Statistical significance was determined by one‐way ANOVA followed by the Tukey–Kramer post hoc test.

Body weight and tissue weights are summarized in Figure 1. WD feeding significantly increased body weight compared with the CON diet. At Week 12, body weight in the WD group was significantly higher than that in the CON group. Two‐way repeated‐measures ANOVA revealed a significant time × group interaction among the four groups, indicating that body weight trajectories differed among groups. Pairwise analyses further showed that body weight trajectories differed between the WD and GT groups and between the WD and SR groups, whereas no significant difference was observed between the GT and SR groups. However, multiple comparisons at individual time points showed a significant reduction in body weight compared with the WD group only in the GT group at Week 12.

Liver weight and adipose depot weights (epididymal, retroperitoneal, intraperitoneal, and mesenteric fat) were higher in the WD group than in the CON group, and these increases were attenuated by both GT and SR. Hind limb muscle mass did not differ among the groups. Cecal content weight was significantly higher in the GT and SR groups than in the CON and WD groups. Food intake was comparable among the WD, SR, and GT groups throughout the intervention. Total energy intake was similar between the WD and GT groups, whereas it was higher in the SR group than in the WD group; nevertheless, feed efficiency was reduced in the SR group (Figure S1). No mortality, overt toxicity, or abnormal behavior was observed in any group during the 12‐week intervention. These results indicate that the effects of SR and GT on adiposity were not attributable to reduced caloric intake.

### 3.2 Effects of SR and GT Supplementation on Lipid and Glucose Metabolism

**FIGURE 2:**
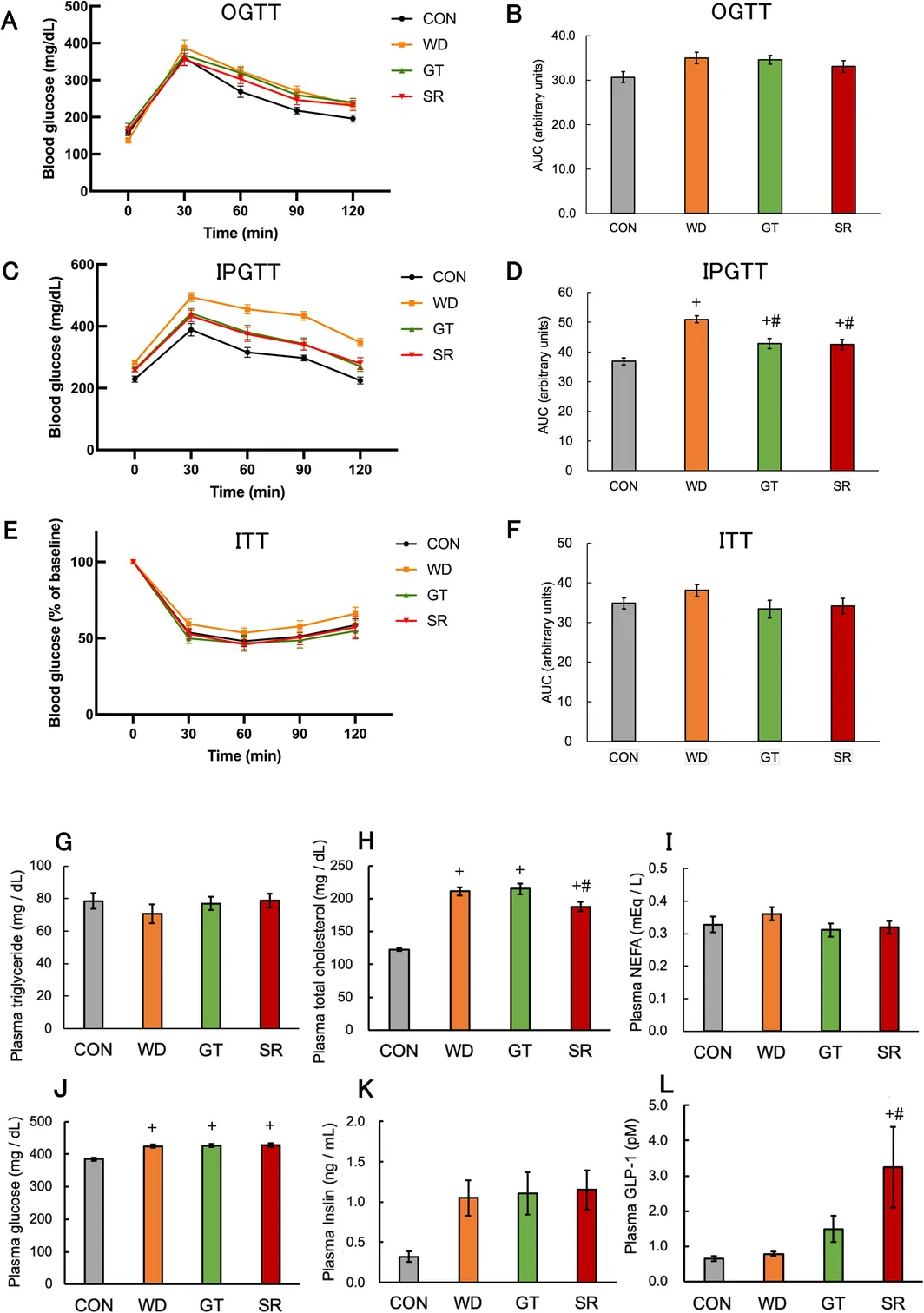
Glucose tolerance/insulin sensitivity and plasma indices. (A, B) Oral glucose tolerance test (OGTT) and areas under the curve (AUC) at Week 9. (C, D) Intraperitoneal glucose tolerance test (IPGTT) and AUC at Week 10. (E, F) Insulin tolerance test (ITT) and AUC at Week 11. (G–L) Plasma concentrations of triglycerides, total cholesterol, nonesterified fatty acid, glucose, insulin, and glucagon‐like peptide‐1 after 12 weeks. Data are presented as mean ± SE (*n* = 9–11), ^+^ *p* < 0.05 versus CON, ^#^ *p* < 0.05 versus WD. Statistical significance was determined by one‐way ANOVA followed by the Tukey–Kramer post hoc test.

**FIGURE 3:**
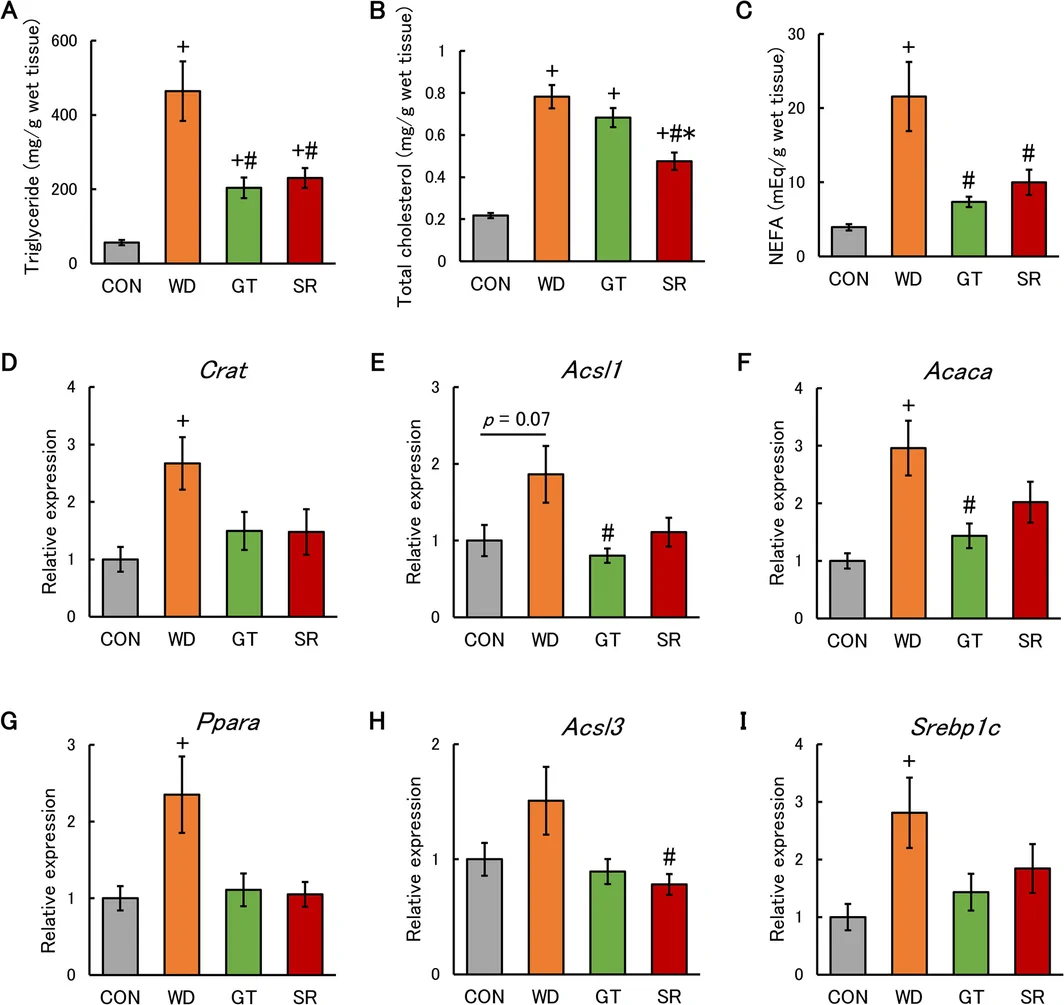
Hepatic lipids and expression of lipid‐metabolism genes. (A–C) Hepatic concentrations of triglycerides, total cholesterol, and nonesterified fatty acid after 12 weeks. (D–I) Hepatic mRNA expression of lipid‐metabolism genes. mRNA levels were normalized to ribosomal protein S18 (*Rps18*) and presented as fold‐change values compared with the control group. Data are presented as mean ± SE (*n* = 9–11), ^+^ *p* < 0.05 versus CON, ^#^ *p* < 0.05 versus WD, *p < 0.05 versus GT. Statistical significance was determined by one‐way ANOVA followed by the Tukey–Kramer post hoc test.

Plasma and hepatic biochemical parameters are shown in Figures 2 and 3. Plasma TG and NEFA concentrations did not differ significantly among the groups. Plasma TC was significantly higher in the WD group than in the CON group, and this increase was significantly attenuated by SR, whereas GT did not reach statistical significance. Fasting plasma glucose was significantly elevated in WD‐fed mice and was not improved by either SR or GT. No significant differences in fasting plasma insulin levels were observed among the groups. Glucose tolerance and insulin sensitivity were evaluated by OGTT, IPGTT, and ITT. No significant improvement was observed in OGTT or ITT outcomes in either the SR or GT group. In contrast, both SR and GT significantly reduced the IPGTT AUC values compared with the WD group, although the time‐course data did not show a significant time × group interaction. Fasting plasma active GLP‐1 levels did not differ between the CON and WD groups but were significantly higher in the SR group than in the WD group. GLP‐1 levels in the GT group were intermediate between those in the WD and SR groups, without reaching statistical significance. WD feeding also elevated hepatic TG and NEFA levels, and both were reduced by SR and GT, with no significant difference between these two groups. In contrast, hepatic TC was significantly lower in the SR group than in both the WD and GT groups. Thus, SR significantly reduced plasma TC compared with WD, while hepatic TC showed a more distinct SR‐associated reduction, with a significant difference from both WD and GT.

### 3.3 Hepatic Expression of Lipid‐Metabolism‐Related Genes

Because SR and GT attenuated hepatic lipid accumulation, we next examined the hepatic expression of lipid‐metabolism‐related genes (Figure 3). WD feeding increased the expression of genes related to fatty acid oxidation (e.g., *Crat* and *Pparα*) and lipogenesis (e.g., *Srebp1c* and *Acaca*). GT significantly attenuated WD‐induced increases in *Acsl1* and *Acaca* expression and tended to reduce the expression of *Crat*, *Pparα*, *Acsl3*, and *Srebp1c*. Similarly, SR significantly reduced *Acsl3* expression and tended to attenuate WD‐induced increases in *Crat*, *Pparα*, *Acsl1*, *Acaca*, and *Srebp1c*. Overall, both SR and GT partially normalized WD‐induced transcriptional changes related to hepatic lipid metabolism.

### 3.4 Cecal Organic Acid Profiles

**FIGURE 4:**
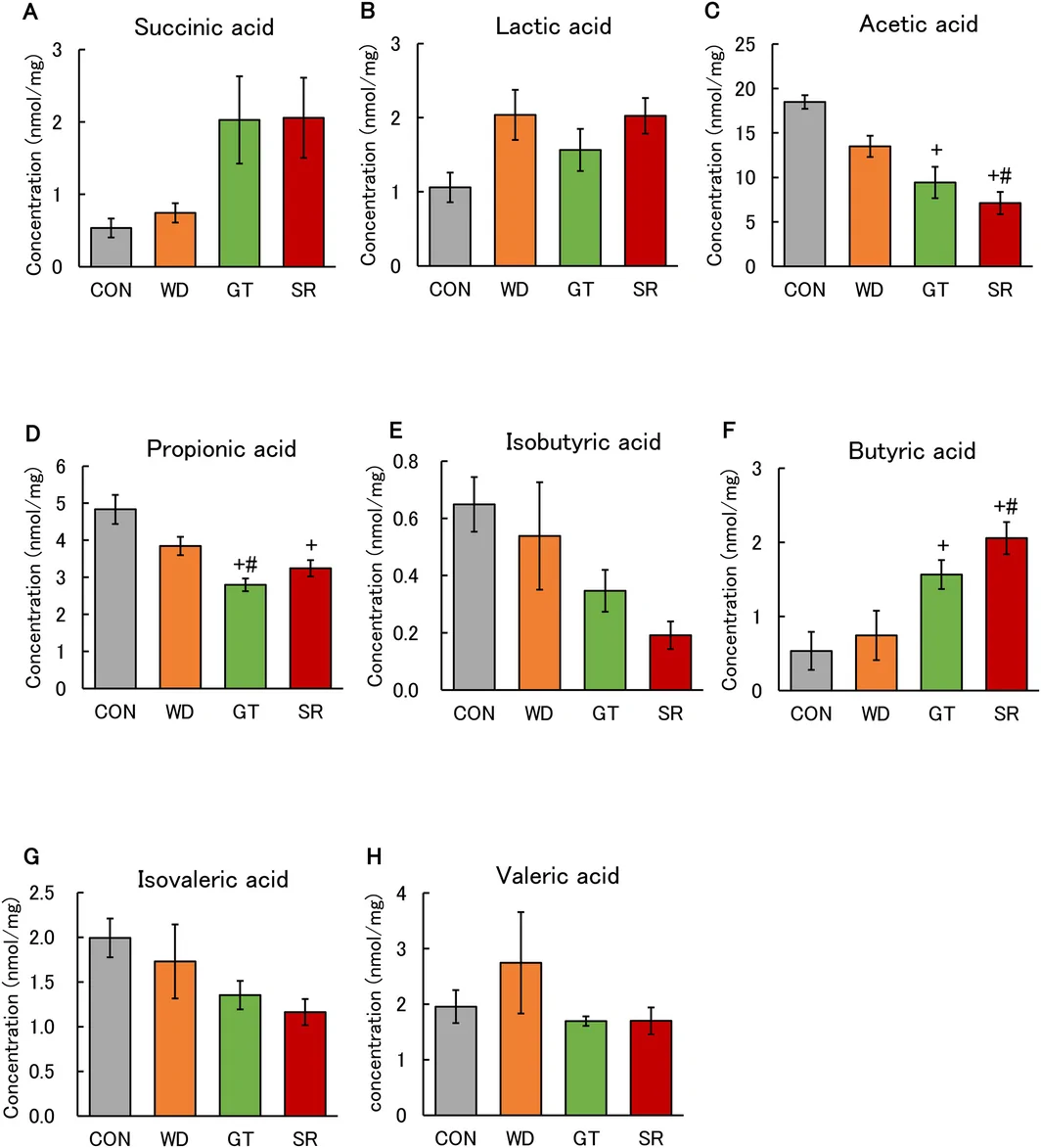
(A) Succinic acid, (B) lactic acid, (C) acetic acid, (D) propionic acid, (E) isobutyric acid, (F) bytyric acid, (G) isovaleric acid, (H) valeric acid Concentrations of organic acids in cecal contents. Concentrations of succinic acid, lactic acid, acetic acid, propionic acid, isobutyric acid, butyric acid, isovaleric acid, and valeric acid in cecal contents were quantified by HPLC. Data are presented as mean ± SE (*n* = 9–10), ^+^ *p* < 0.05 versus CON, ^#^ *p* < 0.05 versus WD. Statistical significance was determined by one‐way ANOVA followed by the Tukey–Kramer post hoc test.

Organic acid concentrations in cecal contents were quantified using HPLC (Figure 4). GT significantly decreased propionic acid concentration, whereas SR significantly increased butyric acid concentration and decreased acetic acid concentration. These results indicate that SR and GT differentially modulated cecal organic acid profiles under WD‐fed conditions.

### 3.5 Intestinal Microbiota Composition

**FIGURE 5:**
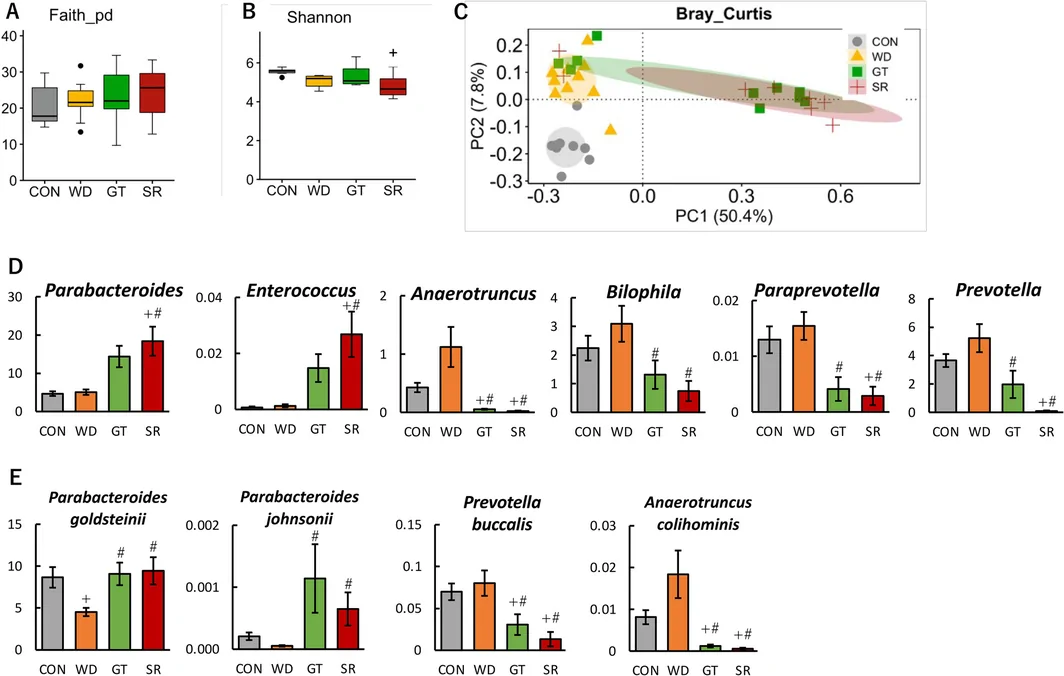
Effects of Sunrouge (SR) and green tea (GT) supplementation on intestinal microbiota composition in Western diet‐fed mice. (A, B) Alpha diversity indices (Faith's phylogenetic diversity, Shannon index). Boxplots show medians and interquartile ranges; dots (●) represent outliers. Statistical significance was assessed by one‐way ANOVA followed by the Tukey–Kramer test. Data are presented as mean ± SE (*n* = 9–10), ^+^ *p* < 0.05 versus CON. (C) Principal coordinates analysis (PCoA) based on Bray–Curtis distances. PC1 and PC2 explained 50.4% and 7.8% of the variation, respectively. PERMANOVA showed a significant overall difference among the four groups (*R* ^2^ = 0.369, *p* = 0.001). (D, E) Relative abundance of representative taxa at genus and species levels. Data are presented as mean ± SE (*n* = 9–10). Taxon‐level comparisons were performed using the Kruskal–Wallis test followed by Dunn's multiple‐comparison test, with *p*‐values adjusted using the Benjamini–Hochberg method. ^+^Adjusted *p* < 0.05 versus CON; ^#^Adjusted *p* < 0.05 versus WD. Adjusted *p*‐values for all pairwise comparisons of the taxa shown in (D, E) are provided in Table S8.

Because cecal organic acid profiles may reflect microbial activity, we next examined intestinal microbiota composition (Figure 5). The number of non‐chimeric reads retained per sample ranged from 39,412 to 166,358, with a mean of 81,338 reads. The number of retained reads for each sample is provided in Table S6. Alpha‐diversity indices, including Faith's phylogenetic diversity and Shannon index, are shown in Figure 5A,B. The Shannon index differed significantly between the SR and CON groups. Beta‐diversity analyses based on Bray–Curtis showed a significant overall difference in intestinal microbiota composition among the four groups (PERMANOVA, *R* ^2^ = 0.369, *p* = 0.001). Pairwise analysis showed significant differences between the CON group and each of the other three groups and between the WD group and both the GT and SR groups, whereas no significant difference was observed between the GT and SR groups (Figure 5C and Table S7).

We next examined representative taxa that showed significant differences after Benjamini–Hochberg correction (Figure 5D,E). At the genus level, *Parabacteroides* and *Enterococcus* were significantly more abundant in the SR group than in the CON and WD groups, whereas these changes were not significant in the GT group. In contrast, *Anaerotruncus*, *Bilophila*, *Paraprevotella*, and *Prevotella* were higher in the WD group than in both the SR and GT groups. At the species level, *Parabacteroides goldsteinii* and *Parabacteroides johnsonii* were significantly more abundant in both the SR and GT groups than in the WD group. Conversely, *Prevotella buccalis* and *Anaerotruncus colihominis* showed higher relative abundances in the WD group than in both the SR and GT groups. LEfSe analysis further identified distinct bacterial taxa associated with each dietary group, supporting diet‐associated differences in intestinal microbiota composition (Figure S2).

### 3.6 Inhibition of Pancreatic Lipase Activity by SR and GT In Vitro

**FIGURE 6:**
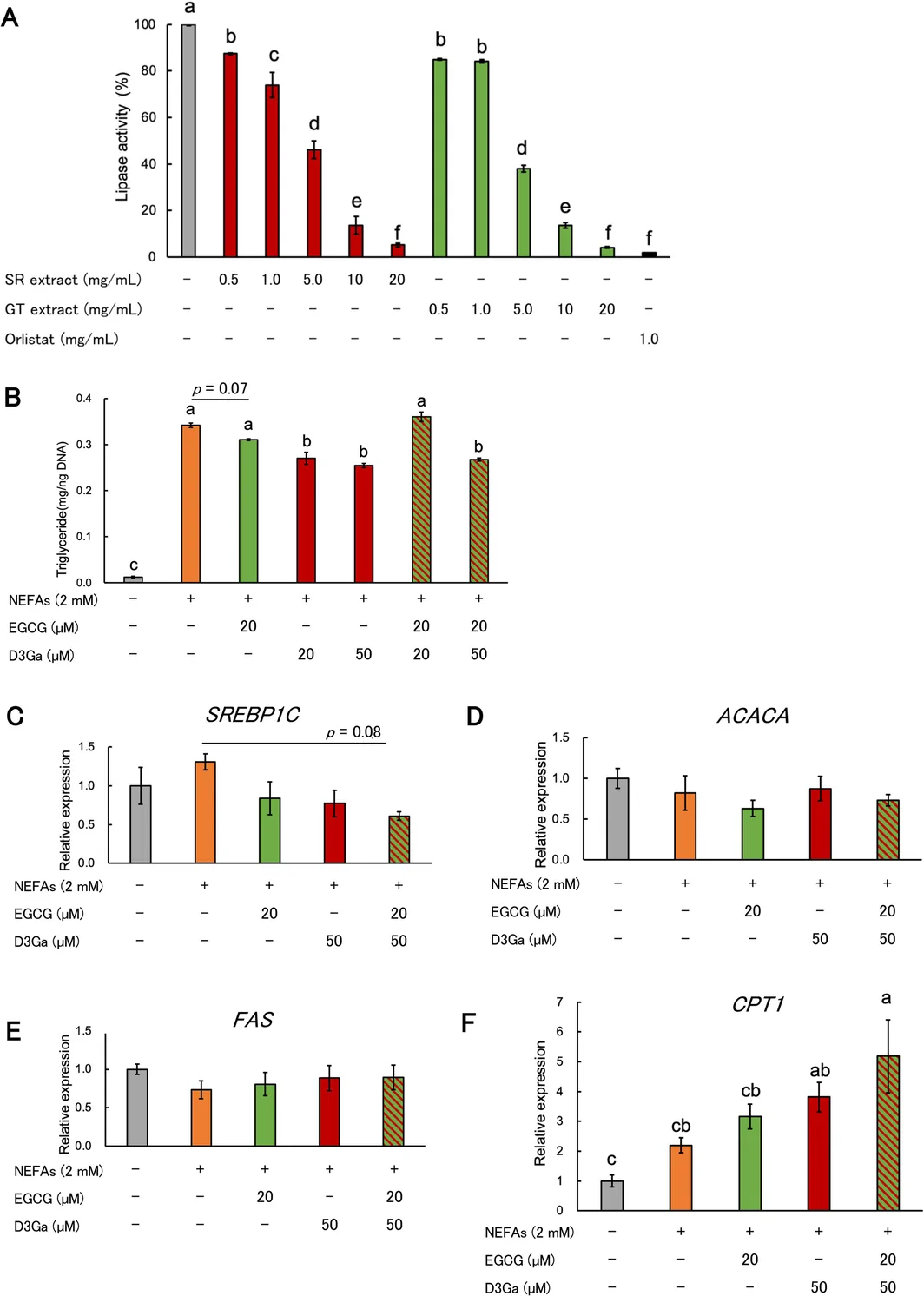
Effects of Sunrouge (SR) and green tea (GT) extracts and their representative polyphenols on pancreatic lipase activity and lipid accumulation in HepG2 cells. (A) Pancreatic lipase inhibitory activity of SR and GT extracts at the indicated concentrations. Lipase activity was measured using Lipase Kit S in vitro. Orlistat (1 mg/mL) was used as a positive control for lipase inhibition. (B) Intracellular triglyceride (TG) levels in HepG2 cells treated with epigallocatechin gallate (EGCG) and/or delphinidin‐3‐*O*‐galactoside (D3Ga) in the presence or absence of nonesterified fatty acids (NEFAs; 2 mM). (C–F) Relative mRNA expression levels of genes involved in lipid metabolism in HepG2 cells treated with EGCG and/or D3Ga. Cells were pretreated with EGCG or D3Ga for 20 h and then incubated with NEFAs for an additional 24 h. Intracellular TG levels were normalized to DNA content. Gene expression levels were normalized to *RPS18*. Data are presented as mean ± SE (*n* = 4). Statistical significance was determined by one‐way ANOVA followed by the Tukey–Kramer post hoc test. Different letters indicate significant differences at *p* < 0.05. N.S. indicates no significant difference (*p* > 0.05).

To investigate a potential mechanism underlying the reductions in adiposity observed in the SR and GT groups, we assessed pancreatic lipase inhibition in vitro (Figure 6A). Both SR and GT significantly inhibited pancreatic lipase activity at concentrations above 0.5 mg/mL. No significant difference was observed between SR and GT at any tested concentration. These results suggest that SR and GT possess comparable pancreatic lipase inhibitory activity.

### 3.7 Effects of EGCG and D3Ga on NEFA‐Induced Intracellular Lipid Accumulation in HepG2 Cells

Because SR and GT altered the hepatic expression of lipid‐metabolism‐related genes in vivo, we next examined the effects of catechins and anthocyanins in HepG2 cells (Figure 6B–F). NEFA treatment significantly increased intracellular TG levels compared with the control, confirming lipid accumulation. Compared with NEFA alone, TG levels tended to decrease with EGCG treatment (*p* = 0.07) and were significantly decreased by D3Ga at 20 and 50 μM. Co‐treatment with 20 μM EGCG and 50 μM D3Ga also significantly reduced TG levels compared with NEFA alone; however, this reduction was not significantly different from that induced by 50 μM D3Ga alone. Among genes related to FA and TG synthesis, *SREBP1C* expression was not significantly altered by EGCG or D3Ga alone but tended to decrease with co‐treatment (*p* = 0.08), whereas *fatty acid synthase* (*FAS*) and *ACACA* expression did not change significantly. Among genes related to fatty acid oxidation, *carnitine palmitoyltransferase I* (*CPT1*) expression was significantly increased by co‐treatment with EGCG and D3Ga, while the effects of the individual treatments were limited. These findings suggest that SR‐derived polyphenols may contribute to the regulation of hepatic lipid metabolism, although clear evidence of synergistic interactions was limited under the present experimental conditions.

## 4 Discussion

In this study, long‐term SR supplementation attenuated several WD‐induced obesity‐related phenotypes in mice. Both SR and GT reduced adipose depot weights and hepatic TG and NEFA levels, and improved glucose excursions in the IPGTT, whereas OGTT, ITT, and fasting plasma glucose were not significantly improved. Notably, SR significantly attenuated the WD‐induced elevation of plasma TC. In addition, hepatic TC was significantly lower in the SR group than in both the WD and GT groups. Although many metabolic effects of SR were shared with GT, SR was additionally associated with lower plasma and hepatic TC, increased fasting active GLP‐1, and distinct alterations in cecal organic acid profiles and gut microbiota composition.

Pancreatic lipase inhibition may be one mechanism contributing to the reductions in adiposity and hepatic lipid accumulation observed in the SR and GT groups. Both tea extracts inhibited pancreatic lipase activity in a dose‐dependent manner (Figure 6A), and this finding agrees with earlier evidence that tea catechins and anthocyanin‐rich extracts can interfere with lipid digestion and absorption (Ikeda et al. 2005; Grove et al. 2012; Chamnansilpa et al. 2020). Reduced intestinal lipid absorption would also be consistent with the attenuation, or tendency toward attenuation, of hepatic expression of genes related to fatty acid synthesis (e.g., *Acaca* and *Srebp1c*) and fatty acid oxidation (e.g., *Crat* and *Pparα*). Food intake was comparable among the WD, GT, and SR groups, whereas total energy intake was higher in the SR group and feed efficiency was lower, suggesting that the SR‐associated reduction in adiposity was not simply attributable to reduced food intake or poor palatability. Cecal content weight was also increased in both the GT and SR groups, indicating that changes in gut contents may have influenced body weight and feed efficiency estimates. Together, these findings suggest that both SR and GT may reduce hepatic lipid accumulation and adiposity, at least partly through reduced intestinal lipid availability and/or altered gut‐related energy handling. However, whether luminal lipase inhibition contributed to the metabolic effects observed in vivo was not directly tested and fecal lipid excretion or apparent energy absorption was not assessed in the present study.

One notable feature of SR was the increase in fasting active GLP‐1, which was not observed in the GT group (Figure 2). Because GLP‐1 has been implicated in cholesterol homeostasis, including regulation of ATP‐binding cassette transporter A1 via miR‐19b (Yao et al. 2018), the elevated fasting GLP‐1 observed in the SR group may be related to its improved TC profile. However, GLP‐1 was measured only in the fasting state in the present study, and post‐load dynamics were not evaluated, because the present study was designed to evaluate basal metabolic parameters after long‐term dietary intervention rather than acute postprandial hormone responses. Further studies, including time‐course analysis and receptor blockade experiments, will be needed to clarify the mechanism underlying this association.

SR and GT also differentially altered cecal organic acid profiles, with SR increasing butyric acid and decreasing acetic acid, whereas GT decreased propionic acid (Figure 4). Because organic acids, particularly butyric acid, can stimulate gut hormone secretion including GLP‐1 and modulate host energy metabolism (Kou et al. 2023), the SR‐associated increase in butyric acid may be related to the GLP‐1 and metabolic phenotypes observed in this study. Consistent with this possibility, both SR and GT altered intestinal microbial composition (Figure 5). Beta‐diversity analysis showed significant differences in microbiota composition among the four dietary groups, and LEfSe analysis further identified bacterial taxa associated with each group. At the genus level, *Parabacteroides* was significantly more abundant in the SR group than in the WD group, while at the species level, *Parabacteroides goldsteinii* was significantly more abundant in both the SR and GT groups than in the WD group. *P. goldsteinii* has been reported to improve obesity‐related metabolic phenotypes (Cui et al. 2022; Wu et al. 2019), suggesting that the increase in *Parabacteroides*‐related taxa may be associated with metabolic effects shared by SR and GT. In contrast, *Bilophila* was more abundant in the WD group than in the SR and GT groups. Because *Bilophila wadsworthia* has been reported to aggravate high‐fat diet‐induced metabolic dysfunctions, including hepatic steatosis and glucose dysregulation (Natividad et al. 2018), the lower abundance of *Bilophila* in the SR and GT groups may reflect a shift away from a WD‐associated microbial profile. Previous studies have proposed that GT may exert metabolic benefits partly through modulation of gut microbiota, including increases in organic acid‐producing bacteria and decreases in inflammation‐associated taxa (Sidhu et al. 2023; Bond and Derbyshire 2019; Zhang et al. 2022). In the present study, SR intake was also associated with a microbiota composition distinct from that of the WD group, and this is the first to characterize such alterations after long‐term SR supplementation. Some of these changes overlapped with those observed in the GT group. These findings suggest that SR may share part of the gut microbiota‐related profile reported for GT.

The SR‐associated reduction in plasma TC and the more distinct reduction in hepatic TC may also be related to changes in the gut microbial and metabolic environment. Previous studies have shown that depletion of gut bacteria can alter bile acid metabolism, including reduced formation and excretion of secondary bile acids and increased bile acid reabsorption, suggesting a link between the intestinal microbiota and cholesterol homeostasis (Miyata et al. 2011). In the present study, SR altered cecal organic acid profiles and intestinal microbial composition, raising the possibility that these changes may be involved in the SR‐associated improvement of cholesterol metabolism through gut–liver interactions. However, bile acid composition, fecal sterol and bile acid excretion, and hepatic genes involved in cholesterol transport and bile acid synthesis were not measured in the present study. Thus, whether the microbiota–organic acid–GLP‐1 pathway contributes to the SR‐associated reduction in TC remains to be tested.

Although the GT and SR diets contained the same amount of tea powder, the estimated intake of tea‐derived polyphenols differed between the two groups. Total catechin intake was higher in the GT group, whereas EGCG intake was higher in the SR group, and anthocyanins were supplied only in the SR group (Tables S1 and S5). These differences may partly explain the overlapping and distinct metabolic effects observed between GT and SR. In addition to catechins and anthocyanins, tea‐derived products contain other bioactive components, including caffeine and L‐theanine. Available product information for the GT powder used in this study reports caffeine and catechin contents of 11.2 and 48 mg per serving (0.8 g), corresponding to approximately 14.0 and 60.0 mg/g powder, respectively. These values provide reference information for the commercial GT product, although they were not obtained from the same powder batch used in the present animal study. Although caffeine was not quantified in the present SR powder batch, previous analyses reported caffeine contents of approximately 24.0–33.9 mg/g dry weight in Sunrouge tea leaves and 28.4 mg/g in Sunrouge tea water extract, comparable to 32.79 mg/g in Yabukita tea water extract (Maeda‐Yamamoto et al. 2012; Nesumi et al. 2012). Caffeine has been reported to increase energy expenditure and fat oxidation, particularly in combination with tea catechins (Hursel et al. 2011). L‐theanine, a characteristic tea‐derived amino acid documented in *Camellia sinensis* (Li et al. 2022), may also be present in SR because SR is derived partly from *C. sinensis* . L‐theanine has been reported to ameliorate diet‐induced obesity, improve glucose tolerance and insulin sensitivity, and reduce plasma lipid levels in high‐fat diet‐fed mice, possibly through the browning of white adipose tissue (Peng et al. 2021). Therefore, caffeine, L‐theanine, and other tea‐derived components may have contributed to the metabolic phenotypes observed in the present study. However, caffeine, L‐theanine, total polyphenols, and other non‐catechin/non‐anthocyanin components were not quantified in the same GT and SR powder batches; therefore, their relative contributions could not be determined.

Possible interactions between catechins and anthocyanins should also be considered. Synergistic effects between tea catechins and other polyphenols have been reported previously. For example, eriodictyol, a flavonoid abundant in citrus fruits, has been reported to act synergistically with GT to suppress body fat accumulation and improve lipid metabolism (Yamashita et al. 2016). In addition, anthocyanins can interact non‐covalently with copigments, including flavonoids and other phenolic compounds, through mechanisms such as π–π stacking, hydrogen bonding, and hydrophobic interactions (Ayvaz et al. 2022; Gençdağ et al. 2022). Because SR contains both catechins and anthocyanins, catechin–anthocyanin interactions may influence the stability, bioavailability, or biological activities of these phytochemicals. However, the formation of catechin–anthocyanin complexes, their stability during digestion, and their bioavailability were not examined in the present study. Therefore, this possibility remains speculative and should be addressed in future studies.

In the HepG2 experiments, EGCG and D3Ga were used as representative SR‐related polyphenols. EGCG was selected as a well‐characterized GT catechin with reported effects on hepatic lipid metabolism and cellular lipid accumulation, whereas D3Ga was selected as a characteristic anthocyanin of SR that is absent from conventional GT. However, ECG was also abundant in both GT and SR powders, and ECG and other catechins may also have contributed to the effects of GT and SR. Because these compounds were not examined in the present HepG2 experiments, their roles remain to be clarified.

In this model, D3Ga significantly reduced NEFA‐induced TG accumulation, whereas EGCG showed a similar but nonsignificant tendency. Co‐treatment with EGCG and D3Ga significantly increased *CPT1* expression, while the effects on *SREBP1C*, *FAS*, and *ACACA* were limited. Taken together, these findings support the possibility that SR‐derived polyphenols may influence hepatic lipid metabolism, possibly in part through effects on fatty acid oxidation. However, clear evidence for synergistic interactions between EGCG and D3Ga was limited under the present conditions. Further investigation of interactions between catechins, anthocyanins, and other food‐derived polyphenols will be needed to clarify how complex tea components contribute to metabolic regulation.

This study has several limitations. First, the animal experiment was conducted using male mice only and did not include a pair‐feeding design; therefore, potential sex‐dependent effects and the contribution of differences in energy intake could not be fully evaluated. Second, GLP‐1 was measured only in fasting plasma samples collected without GLP‐1 stabilizers, and dynamic GLP‐1 secretion in response to nutrient intake or glucose loading was not assessed. Third, bile acid composition, fecal sterol and bile acid excretion, and hepatic genes involved in cholesterol transport and bile acid synthesis were not assessed. Fourth, although SR and GT intake were associated with changes in intestinal microbiota composition, causal microbiome experiments were not performed. Fifth, in the HepG2 experiments, the concentrations of EGCG and D3Ga exceeded typical circulating levels (Di Lorenzo et al. 2021), and D3Ga stability under neutral‐pH culture conditions was not directly assessed. In our preliminary LDH release assay, EGCG did not cause overt cytotoxicity under NEFA‐treated conditions, and similar results were observed for cyanidin‐3‐*O*‐glucoside (Figure S3). However, D3Ga cytotoxicity was not directly assessed under the same culture conditions. The concentrations of EGCG and D3Ga were selected based on previous in vitro studies using anthocyanins, including D3Ga, at concentrations from 10 to 100 μM (Mehmood et al. 2022; Molonia et al. 2020; M. Park et al. 2019; Yu et al. 2020; Zhu et al. 2023). Therefore, the findings from the HepG2 experiments should be interpreted with caution, and future studies should better account for bioavailability, compound stability, and cell viability.

## 5 Conclusion

In conclusion, long‐term SR intake attenuated several obesity‐related phenotypes in WD‐fed mice. Although many effects were shared with GT, SR was additionally associated with lower plasma and hepatic TC, increased fasting plasma active GLP‐1, and distinct changes in cecal organic acid profiles and intestinal microbial composition. These findings suggest that SR attenuates diet‐induced fat accumulation similarly to GT while showing additional associations with cholesterol metabolism, fasting active GLP‐1, and gut‐related parameters.

## Author Contributions

**Kyohei Furukawa:** writing – original draft, formal analysis. **Yongshou Yang:** formal analysis, visualization. **Huijuan Jia:** conceptualization, methodology, writing – review and editing, supervision. **Chika Ando:** formal analysis, writing – original draft, investigation, visualization. **Maki Igarashi:** formal analysis. **Shunichi Someya:** methodology, formal analysis, investigation, validation. **Seigo Yamada:** resources. **Hisanori Kato:** conceptualization, writing – review and editing, supervision.

## Funding

This work was supported in part by the Honjo International Scholarship Foundation.

## Ethics Statement

In this study, animal experiments were approved by the Animal Usage Committee of the Graduate School of Agricultural and Life Sciences, The University of Tokyo (approval no. P21‐016) and were conducted in strict accordance with the relevant institutional guidelines.

## Conflicts of Interest

S.Y. is an employee of AZUMA CHEMICAL Co. Ltd., which provided the Sunrouge (SR) powder used in this study. AZUMA CHEMICAL Co. Ltd. had no role in the study design, data collection, analysis, interpretation of the results, or the decision to publish the manuscript. The other authors declare no conflicts of interest.

## Supporting information


**Table S1:** Major catechins and anthocyanins in green tea and Sunrouge powders. Values are expressed per 100 g of dry powder. The catechin and anthocyanin contents were measured using the same batches of green tea and Sunrouge powders as those used in the animal study. C3G, cyanidin‐3‐*O*‐glucoside; C3Ga, cyanidin‐3‐*O*‐galactoside; D3G, delphinidin‐3‐*O*‐glucoside; D3Ga, delphinidin‐3‐*O*‐galactoside; EC, epicatechin; ECG, epicatechin gallate; EGC, epigallocatechin; EGCG, epigallocatechin gallate.
**Table S2:** Feed composition.
**Table S3:** Primer sequences for mouse genes used for real time RT‐PCR.
**Table S4:** Primer sequences for human genes used for real time RT‐PCR.
**Table S5:** Estimated daily intake of catechins and anthocyanins. Estimated intakes were calculated from the mean food intake over the experimental period, the 3.5% dietary inclusion level of GT or SR powder, and the measured catechin and anthocyanin contents of the same powder batches used in the animal study. Values expressed as mg/kg body weight/day were calculated using the mean body weight over Weeks 0–12.
**Table S6:** Sequencing depth and retained reads for each microbiome sample.
**Table S7:** Pairwise PERMANOVA results for gut microbiota composition based on Bray–Curtis distances.
**Table S8:** Pairwise comparisons for taxa shown in Figure 5D,E.
**Figure S1:** (A) Food intake, (B) total calorie intake, and (C) feed efficiency are shown for control (CON), Western diet (WD), Western diet with 3.5% green tea powder (GT), and Western diet with 3.5% Sunrouge powder (SR). Data are presented as means ± SE (*n* = 9–11). Statistical significance was determined by one‐way ANOVA followed by the Tukey–Kramer post hoc test. Different letters indicate significant differences at *p* < 0.05.
**Figure S2:** Linear discriminant analysis effect size (LEfSe) analysis of intestinal microbiota composition. Differentially abundant bacterial taxa among the CON, WD, GT, and SR groups were identified using LEfSe analysis. Bars indicate linear discriminant analysis (LDA) scores on a log10 scale, and colors indicate the dietary group in which each taxon was enriched.
**Figure S3:** Effects of EGCG and C3G on cytotoxicity in NEFA‐treated HepG2 cells. HepG2 cells were seeded in 24‐well plates at 6.25 × 10^4^ cells/well and cultured in medium containing 10% FBS. At 60%–70% confluence, cells were washed with serum‐free medium and then treated with serum‐free medium containing the indicated concentrations of EGCG or C3G for 20 h. The medium was then replaced with serum‐free medium containing 2 mM NEFAs, and the cells were incubated for an additional 24 h. Cytotoxicity was assessed by measuring lactate dehydrogenase release into the culture medium. (A) EGCG. (B) C3G. Gray bars, untreated control; orange bars, NEFAs alone; green bars, EGCG; yellow bars, C3G; black bars, positive control. Data are presented as the mean ± SE (*n* = 4). Statistical significance was assessed by one‐way ANOVA followed by the Tukey–Kramer test. Different letters indicate significant differences between groups (*p* < 0.05). PC, positive control; 0.2% Tween 20.

## Data Availability

The data supporting the findings of this study are available within the article and its Supporting Information. The raw 16S rRNA gene sequencing data have been deposited in the NCBI Sequence Read Archive under BioProject accession number PRJNA1473370.
