# Supplementary material for: Sunrouge, an Anthocyanin‐Rich Green Tea Cultivar, Attenuates Fat Accumulation and Obesity‐Related Metabolic Abnormalities in Western Diet‐Fed Mice
Source: Food Sci Nutr. 2026 Aug 26;14(8):e72186. doi: 10.1002/fsn3.72186 (PMC13508135; doi:10.1002/fsn3.72186)
Supplement: Supplementary file 1 [file FSN3-14-e72186-s001.docx]

Supplementary Material

# 1 Supplementary Tables

**Supplementary Table S1.** Major catechins and anthocyanins in green tea and Sunrouge powders.

Values are expressed per 100 g of dry powder. The catechin and anthocyanin contents were measured using the same batches of green tea and Sunrouge powders as those used in the animal study.

| g/100 g | Green tea powder | Sunrouge powder |
| --- | --- | --- |
| EC | 0.76 | 0.47 |
| EGC | 3.7 | 1.1 |
| ECG | 4.2 | 3.9 |
| EGCG | 0.70 | 0.92 |
| Total catechins | 9.36 | 6.39 |
| D3Ga | ― | 0.028 |
| D3G | ― | 0.010 |
| C3Ga | ― | 0.014 |
| C3G | ― | 0.004 |
| Total anthocyanins | ― | 0.056 |

Abbreviations: EC, epicatechin; EGC, epigallocatechin; ECG, epicatechin gallate; EGCG, epigallocatechin gallate; D3Ga, delphinidin-3-*O*-galactoside; D3G, delphinidin-3-*O*-glucoside; C3Ga, cyanidin-3-*O*-galactoside; C3G, cyanidin-3-*O*-glucoside.

**Supplementary Table S2.** Feed composition.

**Supplementary Table S3.** Primer sequences for mouse genes used for real time RT-PCR.

| Gene |  | 5'→3' |
| --- | --- | --- |
| *Acaca* | Forward | ATGGGCTGCTTCTGTGACTC |
|  | Reverse | GTTCATCCCTGGGGACCTTG |
| *Acsl1* | Forward | CAGAGGGGGATTCAGGTGTC |
|  | Reverse | GCCAGTTCTGCCACCTCTTT |
| *Acsl3* | Forward | CTATAACGGGGATGGCGAGA |
|  | Reverse | CGGCATCCATGAGAAAGACA |
| *Crat* | Forward | GGAAGAATGGGCTCACACCA |
|  | Reverse | CGGACAGCCAGTTCTCCATT |
| *Ppara* | Forward | TGGAGTCCACGCATGTGAAG |
|  | Reverse | CGCCAGCTTTAGCCGAATAG |
| *Rps18* | Forward | CGTAGTTCCGACCATAAACGATG |
|  | Reverse | GCTATCAATCTGTCAATCCTGTCC |
| *Srebp1c* | Forward | CAGCAGGTCCCAGTTGTACT |
|  | Reverse | GGAGCCAGGGTGCTGATG |

**Supplementary Table S4.** Primer sequences for human genes used for real time RT-PCR.

| Gene |  | 5'→3' |
| --- | --- | --- |
| *SREBP1C* | Forward | TTCCGAGGAACTTTTCGCCG |
|  | Reverse | CCTCGTACGGCCCTTCCTA |
| *ACACA* | Forward | TGTTAAGGCGCTGGTTTGTG |
|  | Reverse | CTCTAGCCACTCCGCCAGAT |
| *FAS* | Forward | AAGGACCTGTCTAGGTTTGATGC |
|  | Reverse | TGGCTTCATAGGTGACTTCCA |
| *CPT1* | Forward | AACATTCCGTACCCCACCAG |
|  | Reverse | CGAATGGGAAGGAATGGAAA |
| *RPS18* | Forward | GGGTGTGGGCCGAAGATATG |
|  | Reverse | TGATCACACGTTCCACCTCA |

| Group | Compound | Content (g/100 g powder) | Estimated intake (mg/mouse/day) | Estimated intake (mg/kg BW/day) |
| --- | --- | --- | --- | --- |
| GT | EC | 0.76 | 0.82 | 27.59 |
|  | EGC | 3.7 | 3.97 | 134.31 |
|  | ECG | 4.2 | 4.51 | 152.47 |
|  | EGCG | 0.7 | 0.75 | 25.41 |
|  | Total catechins | 9.36 | 10.04 | 339.78 |
| SR | EC | 0.47 | 0.55 | 18.40 |
|  | EGC | 1.1 | 1.28 | 43.07 |
|  | ECG | 3.9 | 4.53 | 152.70 |
|  | EGCG | 0.92 | 1.07 | 36.02 |
|  | Total catechins | 6.39 | 7.42 | 250.20 |
|  | D3Ga | 0.028 | 0.03 | 1.10 |
|  | D3G | 0.01 | 0.01 | 0.39 |
|  | C3Ga | 0.014 | 0.02 | 0.55 |
|  | C3G | 0.004 | 0.00 | 0.16 |
|  | Total anthocyanins | 0.056 | 0.07 | 2.19 |

**Supplementary Table S5.** Estimated daily intake of catechins and anthocyanins.

Estimated intakes were calculated from the mean food intake over the experimental period, the 3.5% dietary inclusion level of GT or SR powder, and the measured catechin and anthocyanin contents of the same powder batches used in the animal study. Values expressed as mg/kg body weight/day were calculated using the mean body weight over weeks 0–12.

**Supplementary Table S6**. Sequencing depth and retained reads for each microbiome sample.

**Supplementary Table S7**. Pairwise PERMANOVA results for gut microbiota composition based on Bray–Curtis distances.

| **Group 1** | **Group 2** | **Sample size** | **Permutations** | **Pseudo-F** | **R²** | **p-value** | **BH-adjusted p-value** |
| --- | --- | --- | --- | --- | --- | --- | --- |
| CON | GT | 19 | 999 | 7.390 | 0.303 | 0.0010 | 0.0012 |
| CON | SR | 17 | 999 | 10.556 | 0.413 | 0.0010 | 0.0012 |
| CON | WD | 19 | 999 | 4.679 | 0.216 | 0.0010 | 0.0012 |
| GT | SR | 18 | 999 | 1.355 | 0.078 | 0.2610 | 0.2610 |
| GT | WD | 20 | 999 | 6.517 | 0.266 | 0.0010 | 0.0012 |
| SR | WD | 18 | 999 | 10.042 | 0.386 | 0.0010 | 0.0012 |

**Supplementary Table S8**. Pairwise comparisons for taxa shown in Figure 5D and E.

**Supplementary Figure S1.** (A) Food intake, (B) total calorie intake, and (C) feed efficiency are shown for control (CON), Western diet (WD), Western diet with 3.5% green tea powder (GT), and Western diet with 3.5% Sunrouge powder (SR). Data are presented as means ± SE (n = 9-11). Statistical significance was determined by one‑way ANOVA followed by the Tukey–Kramer post hoc test. Different letters indicate significant differences at *p* < 0.05.

**Supplementary Figure S2.** Linear discriminant analysis effect size (LEfSe) analysis of intestinal microbiota composition. Differentially abundant bacterial taxa among the CON, WD, GT, and SR groups were identified using LEfSe analysis. Bars indicate linear discriminant analysis (LDA) scores on a log10 scale, and colors indicate the dietary group in which each taxon was enriched.

**Supplementary Figure S3.** Effects of EGCG and C3G on cytotoxicity in NEFA-treated HepG2 cells. HepG2 cells were seeded in 24-well plates at 6.25 × 10^4^ cells/well and cultured in medium containing 10% FBS. At 60–70% confluence, cells were washed with serum-free medium and then treated with serum-free medium containing the indicated concentrations of EGCG or C3G for 20 h. The medium was then replaced with serum-free medium containing 2 mM NEFAs, and the cells were incubated for an additional 24 h. Cytotoxicity was assessed by measuring lactate dehydrogenase release into the culture medium. (A) EGCG. (B) C3G. Gray bars, untreated control; orange bars, NEFAs alone; green bars, EGCG; yellow bars, C3G; black bars, positive control. Data are presented as the mean ± SE (n = 4). Statistical significance was assessed by one-way ANOVA followed by the Tukey–Kramer test. Different letters indicate significant differences between groups (*p* < 0.05). PC, positive control; 0.2% Tween 20.
